# Molecular Epidemiology of Female Lung Cancer

**DOI:** 10.3390/cancers3021861

**Published:** 2011-04-01

**Authors:** Seon-Hee Yim, Yeun-Jun Chung

**Affiliations:** 1 Integrated Research Center for Genome Polymorphism, The Catholic University of Korea School of Medicine, 505 Banpo-dong, Seocho-gu, Seoul 137-701, Korea; E-Mail: lyra@catholic.ac.kr; 2 Department of Medical Humanities and Social Sciences, The Catholic University of Korea School of Medicine, 505 Banpo-dong, Seocho-gu, Seoul 137-701, Korea; 3 Department of Microbiology, The Catholic University of Korea School of Medicine, 505 Banpo-dong, Seocho-gu, Seoul 137-701, Korea

**Keywords:** female lung cancer, sex difference, genetic susceptibility, SNP, smoking

## Abstract

Lung cancer is still a leading cause of cancer mortality in the world. The incidence of lung cancer in developed countries started to decrease mainly due to global anti-smoking campaigns. However, the incidence of lung cancer in women has been increasing in recent decades for various reasons. Furthermore, since the screening of lung cancer is not as yet very effective, clinically applicable molecular markers for early diagnosis are much required. Lung cancer in women appears to have differences compared with that in men, in terms of histologic types and susceptibility to environmental risk factors. This suggests that female lung cancer can be derived by carcinogenic mechanisms different from those involved in male lung cancer. Among female lung cancer patients, many are non-smokers, which could be studied to identify alternative carcinogenic mechanisms independent from smoking-related ones. In this paper, we reviewed molecular susceptibility markers and genetic changes in lung cancer tissues observed in female lung cancer patients, which have been validated by various studies and will be helpful to understand the tumorigenesis of lung cancer.

## 1. Introduction

Lung cancer has been the most common cancer in the world for several decades. In 2008, there were an estimated 1.61 million new cases, representing 12.7% of all new cancers and lung cancer was the most common cause of death from cancer with 1.38 million deaths (18.2% of the total) [1]. Lung cancer was still more common in men worldwide (1.1 million cases, 16.5% of the total). However, it became the fourth most frequent cancer of women (513,000 cases, 8.5% of all cancers) and the second most common cause of death from cancer (427,000 deaths, 12.8% of the total) [1]. In Western countries, the incidence and mortality for lung cancer has reached the peak in men, and seems to now be declining. In women, however, incidence and mortality are still approaching the plateau.

Clinically, lung cancer is classified as small cell lung cancer (SCLC) and non-small cell lung cancer (NSCLC). NSCLC accounts for approximately 80% of all cases of lung cancer and is histopathologically subclassified as squamous cell carcinoma (SCC), adenocarcinoma (AC), and large cell carcinoma, for which prognosis and management are similar [2]. In 1984, based on a study involving interviews with 7,804 cases and 15,207 hospital-based controls, Lubin and Blot reported that among those who never smoked there were remarkable cell type differences by sex, with a greater proportion of AC compared to SCC in females (45% *vs.* 25%) than in males (35% *vs.* 33%) [3]. Since then, the incidence of AC has been reported to be increasing in both genders, which has been attributed to changes in the composition of cigarettes and the implementation of filters [4-6]. In 2009, Egleston *et al.* reported the epidemiology of lung cancer using SEER 9-registry data over the past 35 years [7]. AC has remained the most prevalent tumor type in women and its incidence rates have been increasing over time. In contrast, SCC has been predominant in men and its incidence has declined and converged with the rates in women. The rate of large cell carcinoma in women has been similar to that of men, with rates decreasing slightly over time, but SCLC has remained relatively constant in both genders [7]. Due to the confounding effect of smoking and related factors, it is difficult to see whether there is a genuine difference between sexes in histologic types of lung cancer.

There have been many studies that reported the possible higher susceptibility of women to lung cancer compared with men regardless of smoking status. Brownson *et al.* reported higher odds ratios (OR) of ever-smoking and level of smoking in women for all histologic types of lung cancer except AC compared with men based on 14,596 cases and 36,438 age-matched controls [8]. Risch *et al.* reported that a risk of lung cancer increased with smoking in both sexes, but the association was significantly stronger for females than for males in each of the major histologic types [9]. The OR for women with a history of 40 pack-years was 27.9 (95% confidence interval [CI] 14.9–52.0) and that for men was 9.6 (95% CI 5.6–16.3) compared with lifelong nonsmokers [9]. Harris *et al.* also reported that women are at higher risk than men for a given level of smoking (OR 1.7, 95% CI 1.2–2.2) [10]. Zang and Wydner reported that dose-dependent ORs over cumulative exposure to cigarette smoking were 1.2-fold to 1.7-fold higher in women than in men for the three major histologic types (squamous/epidermoid, large-cell, and AC type) and concluded that this gender difference cannot be explained by differences in baseline exposure, smoking history, or body size, but it is more likely due to the higher susceptibility to tobacco carcinogens in women [11].

However, there are studies that have reported conflicting results. In a large prospective population based study (30,874 subjects included, 422,606 person-years of observation, 867 new lung cancer cases), Prescott *et al.* found that incidence rates of lung cancer among female and male never-smokers were similar and, after being adjusted for pack-years, age, and study population, the rate ratio between female and male smokers was 0.8 (95% CI 0.3–2.1), which means the incidence rates were also similar in smokers regardless of sex [12]. Bain *et al.* analyzed prospective data from former and current smokers in two large cohorts—the Nurses' Health Study and the Health Professionals Follow-up Study—and calculated incidence rates and hazard ratios of lung cancer in women compared with men [13]. After adjusting for age, number of cigarettes smoked per day, age at start of smoking, and time since quitting, the hazard ratio in women ever smokers compared with men was 1.11 (95% CI 0.95–1.31), which suggests that women do not seem to have a greater susceptibility to lung cancer than men, given equal smoking exposure [13]. Studies reporting higher susceptibility of women to lung cancer are mostly case-control studies, which are well known to be vulnerable to various biases. In contrast, two studies reporting null results are based on large prospective cohorts. Furthermore, exposure measurement cannot be said to be objective and accurate, because most studies used questionnaires for exposure measurement. It is necessary to apply biomarkers for accurate exposure measurement to determine whether the susceptibility to lung cancer is really different between sexes.

Apart from incidence, gender disparities have been observed in cancer progression, survival, and therapeutic response [14]. A variety of mechanisms, biological, behavioral and environmental, should be behind these differences. Oncogenesis is a complex, multifactorial process which involves various factors; host factors such as xenobiotic metabolism, sex, age, and hormones; and environmental factors such as tobacco smoke, asbestos, diet, and air pollution, *etc.* In this review, we examined germ-line genetic polymorphisms and somatic alterations in cancer tissues as host factors, especially ones distributed differently between sexes, which may explain clinical differences in female and male lung cancer.

## 2. Sex-specific Characteristics

### 2.1. Metabolism-Related Genetic Polymorphisms

Cigarettes contain a mixture of carcinogens, including a small dose of polycyclic aromatic hydrocarbons (PAHs) and 4-(methylnitrosamino)-1-(3-pyridyl)-1-butanone (NNK). Carcinogens such as NNK and PAHs require metabolic activation to exert their carcinogenic effects; there are competing detoxification pathways, and the balance between metabolic activation and detoxification differs among individuals and will affect cancer risk. Many (pro)carcinogens are converted to biologically reactive metabolites by Phase I enzymes such as those encoded by the cytochrome P450 (CYP) super family (CYP1, CYP2, CYP3, and CYP4 isoforms included) [15]. The metabolic activation process leads to the formation of DNA adducts, which are carcinogen metabolites bound covalently to DNA [2,16]. If DNA adducts escape cellular repair mechanisms and persist, they may result in permanent mutations and initiate carcinogenesis. If a permanent mutation occurs in a critical region of an oncogene or tumor suppressor gene, it can lead to activation of the oncogene or deactivation of the tumor suppressor gene. Multiple events of this type lead to aberrant cells with loss of normal growth control and, ultimately, to lung cancer [17]. Following the Phase I reaction, Phase II enzymes such as glutathione S-transferases (GSTs) are responsible for detoxifying the activated forms of PAH epoxides. The GSTs also form a supergene family and the major isoforms are *GSTM1, GSTM3, GSTT1*, and *GSTP1* [18]. Genetic polymorphisms have been found in both Phase I and Phase II enzymes and some of them show sex-specific differences in the effect. Many papers have been reporting the association between polymorphisms and lung cancer, but a few of them analyzed differences in the effects of polymorphisms on lung cancer risk between sexes.

Tang *et al.* reported based on a case-control study that *GSTM1* null genotype (inherited deletion) was significantly associated with NSCLC (OR 2.04, 95% CI 1.13-3.68) [19]. The ORs for *GSTM1* null genotype were significant in female smokers (OR 3.03, 95% CI 1.09–8.40) and not in males (OR 1.42, 95% CI 0.53–4.06), which suggests that the effect of the *GSTM1* null genotype on NSCLC is greater in female smokers than in male smokers with an equal smoking history [19]. Dresler *et al.* genotyped *GSTM1* and *CYP1A1* (Ile462Val, rs1048943) in a case-control study and found that although the OR for the *CYP1A1* polymorphism in females and males were not significantly different, the effect of the *CYP1A1* polymorphism may be stronger in female than in male smokers (OR 4.98 in women, 95% CI 1.50-16.39 *vs.* 1.37 in men, 95% CI 0.44–4.31). Although lung cancer risk did not increase significantly for *GSTM1* null for either sex in the multivariate analysis, the effect of the *CYP1A1* polymorphism may be stronger in *GSTM1* null females (OR 6.54 in women, 95% CI 1.1–40.0 *vs.* 2.36 in men, 95% CI 0.49–11.49), independent of age or smoking history. These data suggest that polymorphisms in *CYP1A1* and *GSTM1* contribute to the increased risk of females for lung cancer [20]. In 2006, one meta-analysis of 130 studies (over 23,000 cases and 30,000 controls) reported the weakly positive, but not significant association between the *GSTM1* null genotype and the risk of lung cancer [21]. Assuming that this conclusion is true, it will also be far more difficult to identify the sex-specific effect of *GSTM1* polymorphism.

Hung *et al.* performed a pooled analysis of 14 case-control studies on lung cancer in Caucasian non-smokers and reported the OR of lung cancer for the *CYP1A1* Ile462Val polymorphism was 2.99 (95% CI 1.51–5.91) [22]. The gender-specific ORs were 3.52 (95%CI 1.35–9.17) for men and 2.57 (95% CI 0.58–11.5) for women, which is different from the ORs from Dresler *et al.* Furthermore, the OR for women did not achieve statistical significance. Le Marchand *et al.* carried out a pooled analysis of the data submitted to the Genetic Susceptibility to Environmental Carcinogens (GSEC) database [23]. They reported that the pooled ORs for *CYP1A1* Ile462Val heterozygote and homozygote polymorphisms were 1.15 (95% CI 0.95–1.39) and 1.54 (95% CI 0.97–1.46), respectively, and that the *CYP1A1* Ile462Val polymorphism may confer an increased risk of lung cancer in women (OR 2.70, 95% CI 1.79–4.08 *vs.* OR 1.08, 95% CI 0.88–1.32 in men) [23]. Timofeeva *et al.* conducted a case-control study (638 Caucasian patients under the age of 51 with primary lung cancer and 1,300 cancer-free control individuals) and examined the effect of 13 polymorphisms in the *CYP1A1, CYP1B1, CYP2A13, CYP3A4* and *CYP3A5* genes [24]. No significant association was found between any of the analyzed polymorphisms and lung cancer risk overall. However, among women, a significantly increased risk was observed for carriers of the minor allele of *CYP1B1* SNP rs1056836 (OR 1.97, 95% CI 1.32–2.94) and the effect was shown to be modified by smoking. The results suggest that the *CYP1B1* polymorphism may contribute to increased susceptibility to early-onset lung cancer in women [24]. Recently, one meta-analysis suggested the protective effect of *CYP2E1* RsaI/PstI and DraI polymorphism for lung cancer, but there is no information on frequency of the polymorphism by sex [25].

Regarding the GSTP1 polymorphisms, a lot of studies have been reporting inconsistent results. Among the studies, four meta- or pooled analysis studies have reported no significant association between the GSTP1 polymorphisms GSTP1 exon 5 (Ile105Val) and the risk of lung cancer and have not provided the gender-specific information [21,26-28]. Some studies have suggested the existence of differences by ethnicity, age of diagnosis, smoking status, and gender in the effect of the GSTP1 polymorphisms on the risk of lung cancer, but evidence is far from enough to draw any conclusions [29-34].

Myeloperoxidase (*MPO*) is a phase I metabolic enzyme that converts the metabolites of benzo[a]pyrene from tobacco smoke into highly reactive epoxides. A polymorphism in the promoter region of MPO (463G>A) has been found to be inversely associated with lung cancer and differences in the association with age and sex have been suggested. Taioli *et al.* conducted a pooled analysis (3,688 cases and 3,874 controls) and found the OR for lung cancer was 0.88 (95% CI 0.80–0.97) for the A/G variant of *MPO* 463G>A polymorphism, and 0.71 (95% CI 0.57–0.88) for the A/A variant after adjusting for smoking, age, gender, and ethnicity [35]. The inverse association between lung cancer and *MPO* 463G>A polymorphism was observed equally in males and females in a different study. However, more recent studies have been reporting no association between polymorphism and lung cancer [36,37].

Wang *et al.* screened common variants in 5′ flanking and 3′ untranslated regions of the adenosine triphosphate-binding cassette B1 (*ABCB1*) and *ABCC1* candidate transporter genes which are the genetic components in the metabolism and disposition of NNK in Chinese population, and examined the association with lung cancer [38]. Compared with the wild A/A genotype, *ABCB1* rs3842 A/G or G/G (OR 1.36, 95% CI 1.06–1.76) and ABCC1 rs212090 A/T or T/T (OR 1.37, 95% CI 1.03–1.83) genotypes were associated with an increased risk of lung cancer. The association of *ABCB1* rs3842 with the risk of lung cancer was stronger in women (OR 2.57, 95% CI, 1.36–4.85) than in men (OR 1.19, 95% CI 0.89–1.58), but *ABCC1* did not show sex difference [38].

Reported polymorphisms associated with female lung cancer risk are listed in the Table 1.

### 2.2. DNA Repair-Related Genetic Polymorphisms

DNA repair capacity (DRC) is an important host factor that may influence lung carcinogenesis. Tobacco smoke contains many carcinogens and reactive oxygen species that produce DNA adducts, cross-links, DNA damage and DNA strand breaks requiring repair through multiple pathways, including the following: base excision repair, nucleotide excision repair, mismatch repair, single-strand break and double-strand break mechanisms. A positive and consistent association was observed between the reduced DRC and cancer occurrence (ORs in the range of 1.4–75.3) [39]. It has been reported that DRC is relatively lower in female lung cancer patients than in male patients [40] and a significant sex difference in antibodies to oxidative DNA damage from cigarette smoking has also been observed [32]. Given the same duration of exposure, women had a higher level of antibodies to oxidative DNA damage and also reached peak levels at a lower cumulative smoking exposure (30 years) compared with male smokers (40 years) [41].

O-alkylated bases, such as O6-methyguanine, are the major carcinogenic lesions in DNA induced by alkylating mutagens. O6-methylguanine, a methylated damage lesion in DNA, correlates with spontaneous G:C>A:T transition mutations and leads to activation of oncogene *K-ras* or dysfunction of the tumor suppressor gene *TP53*. This DNA adduct is removed by the repair protein, O(6)-methylguanine-DNA methyltransferase (*MGMT*) [42]. *MGMT* promoter methylation is a common event in primary human neoplasms including lung cancer [43-46]. The highest prevalence of *MGMT* promoter methylation was found in male nonsmokers followed by male smokers and female nonsmokers, which does not support the hypothesis that the lung cancer risk is higher in women [47]. Although the possible association between *MGMT* polymorphisms and lung cancer risk has been examined in several studies, most results have been inconclusive and not included information on sex difference [48-53]. Only one study reported the sex difference in the effect of *MGMT* polymorphisms. Wang *et al.* examined whether genetic variants of *MGMT* are associated with increased lung cancer risk in a case-control study consisting of 1,121 Caucasian lung cancer patients and 1,163 matched cancer-free controls [54]. They genotyped four potentially functional SNPs of *MGMT*: exon 3 codon 84C>T (L84F), exon 5 codon 143A>G (I143V), and two promoter SNPs 135G> T and 485C>A. They categorized the *MGMT* genotypes as either 0 variants (84CC-143AA) or 1–4 variants. Compared with 0 variants, those with 1–4 variants showed a significantly increased risk of lung cancer (OR 1.19, 95% CI 1.01–1.41) and this increased risk was more prominent in women (OR 1.31, 95% 1.03–1.66) than in men (OR 1.09, 95% CI 0.86–1.83) [54].

Hung *et al.* examined the associations between lung cancer risk and 18 polymorphisms in 12 DNA repair genes, including *APEX1, OGG1, XRCC1, XRCC2, XRCC3, ERCC1, XPD, XPF, XPG, XPA, MGMT, and TP53* [55]. Two of the variants were found to be significantly associated with lung cancer risk; *XRCC3* T241M (heterozygote OR, 0.89, 95% CI 0.79–0.99 and homozygote OR 0.84, 95% CI 0.71–1.00), and *XPD* K751Q (heterozygote OR 0.99, 95% CI 0.89–1.10 and homozygote OR 1.19, 95% CI 1.02–1.39), but there was no sex differences in the stratified estimates of *XRCC3* T241M [55].

### 2.3. Cell Proliferation-Related Genetic Changes

There are many signaling pathways known to be involved with lung carcinogenesis. Multistep accumulation of genetic alterations occurs at the genomic level, leading to initiation, development and maintenance of lung cancer. We focused on genetic polymorphisms and somatic DNA alterations in the genes which have been frequently suggested to be associated with lung cancer.

#### 2.3.1. KRAS

*KRAS* gene encodes an oncogenic protein when mutated or overexpressed, which is known to act as signaling switches that relay growth signals from the cell surface to the mitogen-activated protein (MAP) kinase cascade. Mutations in *KRAS* are specific to AC, and occur rarely in SCC or SCLC. Most *KRAS* mutations in lung AC are smoking-related G to T transversions and affect exon 12 or exon 13, suggesting that *KRAS* mutations are induced primarily by tobacco smoke carcinogenesis [56,57].

There have been studies reporting no association between the *KRAS* point mutation in lung cancer tissue and sex [58-60]. However, Nelson *et al.* reported a significant association between female sex and *KRAS* mutation in lung AC tissue after adjustment for carcinogen exposures (OR 3.3, 95% CI 1.3–7.9) and mutations were found only in smokers [61]. They suggested a possible role of estrogen exposure in either the initiation or the selection of *KRAS* mutant clones in AC [61]. Boldrini *et al.* also reported that among 411 lung AC patients *KRAS* mutations were more frequent in males (23.7% *vs.* 10.1%, P = 0.0007) [62]. More studies will be required to verify the association between *KRAS* mutations and sex.

#### 2.3.2. TP53

Malfunction of the p53 pathway is a phenomenon observed in most human tumors. Somatic mutation of *TP53* is one of the most common mechanisms by which the p53 pathway is damaged during tumorigenesis. Although several studies have reported on sex difference in *TP53* mutations, the results are inconsistent in terms of frequency and mutation types.

Chiba *et al.* examined mutations changing the p53 coding sequence from 51 NSCLC. Mutations were found in 45% of tumor specimens [63]. In multivariate analysis the presence of *TP53* mutations was associated with younger age and squamous histology, but not with tumor stage, nodal status or sex [54]. Kure *et al.* also investigated sex differences in *TP53* mutations from the tumor tissue of NSCLC patients and levels of DNA adducts in non-tumor lung tissue from the patients [64]. Although *TP53* mutations seemed to be more frequent in male patients (53% *vs.* 36%), G:C>T:A mutations were more frequent in females (40%) than in male patients (28%). However, these differences were not statistically significant [64].

One meta-analysis reported that somatic *TP53* gene mutations were observed more often in males than in females (OR 1.59, 95% CI 1.16–2.19) [65]. However, this result could be confounded by the fact that males have relatively more SCCs, which have a higher proportion of *TP53* mutations than do ACs, which occur more frequently in women. They could not determine whether *TP53* gene mutations are associated with sex independently of histologic types. There was no information on the spectrum of *TP53* mutation types, either [65]. Using the International Agency for Research on Cancer *TP53* database, Toyooka *et al.* reported the significant differences in *TP53* mutational spectra in lung cancer tissue between male and female never-smokers [66]. The G:C>T:A transversions were significantly frequent in female smokers (36%) than in female never-smokers (11%), but there was no such difference in the mutational spectra of male never-smokers (31%) and smokers (27%). A very similar pattern was found in the analysis limited to AC. In other words, cancers arising in women smokers seemed to obtain significantly more tobacco-related mutations, which may contribute to the higher susceptibility of women to tobacco carcinogens [66].

Marrogi *et al.* reported a similar frequency and type of somatic *TP53* mutations between men and women based on a case series (102 women and 201 men). The percentage of *TP53* G:C>T:A mutations in women (41%) was slightly higher than in men (38%), but this was not statistically significant [67]. A sex difference in *TP53* somatic mutations seems to exist, but more studies will be required to confirm the association between *TP53* mutations and sexes.

Besides the somatic mutations, numerous SNPs and other sequence variations have been reported at the *TP53* locus. However, there is no report on sex difference in *TP53* polymorphisms. Fang *et al.* reported a 58% higher lung cancer risk in *TP53* germ-line mutation carriers with the *MDM2* SNP309 GG+GT alleles compared with TT homozygotes [68]. *MDM2* SNP309 was located in the promoter of the *MDM2* gene and result in higher levels of *MDM2* RNA and protein, which consequently induce the attenuation of the p53 pathway [69]. A significant effect of SNP309 G allele was observed in women (OR 1.60, 95% CI 1.08–2.36) but not in men (OR 1.47, 95% CI 0.82–2.60), which may be related to biological regulation of *MDM2* by estrogen [68]. In germ-line *TP53* mutation carriers, SNP309 was reported to accelerate tumor onset and to be associated with the development of multiple primary tumors [70,71]. Bond *et al.* also showed that the same polymorphism accelerated tumor formation in women, but not in men and depended upon estrogen signaling [72].

#### 2.3.3. EGFR

Epidermal growth factor receptor (EGFR) plays an important role in the development and progression of a variety of malignant tumors and mutated *EGFR* status is a known predictor of response to tyrosine kinase inhibitors (TKIs). The *EGFR* mutations are distributed throughout the kinase domain, but a deletion in exon 19 and the point mutation L858R in exon 21 account for approximately 90%, which confer a greater response to gefitinib treatment, compared with other types of *EGFR* mutations. There have been many reports on the sex-specific incidence or prevalence of *EGFR* mutations and the results are inconsistent.

Marchetti *et al.* examined somatic *EGFR* mutations in 860 NSCLC patients and found *EGFR* mutations to be independently associated with female sex (OR 2.9, 95% CI 1.3-6.7) [73]. Another study on 35 Taiwanese AC patients also reported a very strong association between somatic *EGFR* mutations and female sex (OR 10.9, 95% CI 1.8-67.0) [74]. Shigematsu *et al.* reported that the frequency of somatic *EGFR* mutations was greater for females *versus* males (42% *vs.* 14%, P < 0.001), never smokers *versus* ever smokers (51% *vs.* 10%, P < 0.001), and for AC *versus* other histologic types (40% *vs.* 3%, P < 0.001) and that results of multivariate analyses confirmed these findings [75]. There are more studies which reported more frequent somatic *EGFR* mutations in women, but most results were based on simple comparison between sexes, which makes it hard to draw meaningful conclusions [62,76-81].

There have been studies which reported no sex difference in *EGFR* mutations. Toyooka *et al.* examined the *EGFR* mutational spectrum in exons 18 to 21 in tumor tissue from 1,467 NSCLC patients [82]. In never smokers, there was no difference between female and male cases. In ever smokers, exon 19 mutations were significantly less frequent in male compared with female cases (OR 0.34, 95% CI 0.16–0.70). In the analysis restricted to ACs, similar results were obtained; in ever smokers, exon 19 mutations were significantly less frequent in males (OR 0.32, 95% CI 0.15–0.67). This finding suggests both sex and smoking status could influence the somatic *EGFR* mutational spectrum [82]. Tanaka *et al.* analyzed the *EGFR* mutation analysis from 1,176 Japanese NSCLC patients and reported that the frequency of *EGFR* mutation was significantly higher in AC (OR 3.18, 95% CI 1.39–7.23) and in light-smokers (OR 3.84, 95% CI 1.92–7.65), but not associated with sex in multivariate analysis [83]. Although the frequency of EGFR mutations seemed to be higher in females (P < 0.0001 for a chi-square test), after adjusting effects of age, sex, histology, staging and smoking history through logistic regression, only the deletions in exon 19 were found to be more frequent in males (P = 0.0011 from logistic regression). It suggested that reportedly more frequent *EGFR* mutations in females may be a reflection of a higher frequency of AC in females [83]. To confirm the existence of the association between sex and the frequency of somatic *EGFR* mutations, it is necessary to perform more detailed studies which can consider potential confounding factors such as tumor differentiation. Together with somatic mutations, increased *EGFR* gene copy number in tumor emerged as another important predictor for TKI sensitivity [84]. However, in various clinical trials, the frequency of *EGFR* gene amplification was not significantly different between sexes [76,85-88].

There are reports on germ-line polymorphisms and other variants in *EGRF*. To explore the association between germ-line polymorphisms of the EGFR and the lung cancer susceptibility, Jou *et al.* genotyped 14 SNPs in *EGFR* and found that a SNP 8227G>A (rs763317) showed statistically significant difference between lung cancer patients and control subjects, and the AA genotype of 8227G>A polymorphism had a significantly increased risk of developing lung cancer compared with the G/G genotype (OR 2.40, 95% CI 1.34–4.33) [89]. Although there was no statistically significant differences between the genotype distribution of *EGFR* 8227G>A polymorphism and cancer histology within the male population, in female population, ORs for 8227G>A were significantly increased in AC subtype (OR for G/A genotype 1.23, 95% CI 0.87–1.75; OR for A/A genotype 3.52, 95% CI 1.32–9.37), but not that in SCC. Haplotype analyses revealed that haplotype comprising the rare allele of 8227G>A, and the common allele of the other 13 SNPs, was associated with a significantly increased risk of female AC (OR 2.81, 95% CI 1.02–7.77) [89]. This result suggests that polymorphisms and haplotypes of the *EGFR* affect the development of lung cancer differently according to sex or histologic types.

## 3. Conclusions

There are many studies which reported sex-specific genomic characteristics, both constitutional and somatic, in the risk of lung cancer, but some of the studies seem to be vulnerable to various biases and confounding factors, which makes the whole picture somewhat confusing. Recent meta-analysis results have reflected this point; quite a few of the previously reported associations between polymorphisms and the risk of lung cancer have been negated. Many studies used information on histologic types and smoking status to adjust their confounding effects, but it is far from complete. Especially smoking status was determined by various methods based on different criteria. To confirm the existence of sex difference in lung cancer, it would be necessary to perform more detailed studies which consider potential confounding factors such as tumor differentiation and stage. It is far from complete to only include histologic types and basic smoking status.

## Figures and Tables

**Table 1.** Reported polymorphisms associated with female lung cancer risk.

**Functions**	**Genes**	**Polymorphisms**	**Remarks**	**Ref.**
Metabolism	CYP1A1	rs1048943,	Higher risk in female non-smoker	[20,22]

	CYP1B1	rs1056836	Higher risk in female smoker	[24]
		rs1056827	Higher risk in female smoker	
		rs2567206	Higher risk in female smoker	

	CYP2A13	rs8192784	Higher risk in male non-smoker	[24]
		rs1709084	Higher risk in female non-smoker	

	GSTM1	Null type	Higher risk in female and smoker	[19]

	ABCB1	rs3842	Higher risk in female	[38]

DNA repair	MGMT	rs1711646, rs1625649, rs12917, rs2308321	Higher risk in female	[54]

Cell proliferation	EGFR	rs763317	Significantly associated with AC in female but not in male	[89]
